# Recidivism treatment manuals: a corpus-based examination for public offender counselors

**DOI:** 10.3389/fpsyg.2026.1564018

**Published:** 2026-03-19

**Authors:** Carlos Obed Texidor Maldonado, Cass Dykeman

**Affiliations:** 1 Western Oregon University, Monmouth, OR, United States; 2 Oregon State University, Corvallis, OR, United States

**Keywords:** collocation, corpus linguistics, evidence-based practices, GraphColl, keyness, manualized treatment, offender counseling, recidivism

## Abstract

**Introduction:**

This study explored two widely used recidivism-prevention manuals: the Cognitive Behavioral Interventions–Core Adult (CBI-CA) and Thinking for a Change (T4C). The aim was to identify key linguistic patterns, including keyness (word-usage differences) and collocation (word-usage associations), that may influence treatment efficacy and cultural responsiveness in offender counseling.

**Measures and methods:**

Keyness analysis, performed via the log-likelihood ratio test (G2), revealed statistically significant lexical differences. Collocation analysis via Mutual Information (MI) highlighted important word-usage associations.

**Results:**

The findings indicated that the CBI-CA manual emphasized motivational and psychoeducational language (e.g., “module,” “success,” and “worksheet”), whereas the T4C manual displayed a more institutional focus (e.g., “lesson,” “supplement,” and “handout”). Collocation analysis further revealed distinct framing of criminogenic factors, with CBI-CA underscoring mental health and personal growth and T4C centering on systemic and justice-related constructs.

**Discussion:**

These findings highlight the need for culturally attuned and linguistically sensitive approaches in evidence-based recidivism treatment. Implications include refining curriculum design to enhance both therapeutic efficacy and inclusivity. Future research should explore the cross-cultural adaptability of treatment manuals to address systemic inequities in offender rehabilitation.

## 1 Introduction

In their song “Locked Up,” Akon and Styles P give voice to the despair of countless Black individuals trapped in the unforgiving cycle of incarceration (Akon and Styles, 2004). In this excerpt from the song, lyricist Akon captures this reality:

*Locked up, they will not let me out*

*And I had a long day in court, shit stress me out*

*Won’t give me a bail, they cannot get me out*

*I used to living luxurious, I do not wanna live here*

This cycle is tragically common. Of those released from incarceration, nearly two-thirds reoffend within a short time. The justice system has a revolving door; an “estimated 68% of persons released from prison were arrested within 3 years, 79% within 6 years, and 83% within 9 years” (U.S. Department of Justice, 2022). While getting out of incarceration is not the problem, the challenge lies in helping individuals stay out. Latessa and Schweitzer (2020) stressed that the ineffectiveness of community supervision contributes to high incidences of recidivism. Incarceration rates reflect racial disparities, with individuals of color incarcerated at “five and a half times the rate of non-Hispanic White males” (Enders et al., 2018, p. 366; Alexander and West, 2012). From a behavioral health perspective, incarceration and recidivism rates are a social justice imperative for clinicians and counselor educators that require purposeful academic research and clinical practices (Chang et al., 2010). The therapeutic interventions presented by recidivism counselors have the potential either to empower individuals to reintegrate successfully into society or perpetuate cycles of incarceration. This study addresses the urgent need to refine these interventions by analyzing the linguistic structures within treatment manuals, emphasizing their role in reducing systemic inequities and improving rehabilitation outcomes.

This study has a twofold purpose: first, to fill gaps in the existing literature on recidivism treatment—specifically, the nature of the discourse in widely used treatment manuals. Second, to disrupt current practice by identifying conceptual holes, cultural blind spots, and pejorative language that may be present in this discourse. As mentioned, BIPOC (i.e., Black, Indigenous, and people of color) men are incarcerated at five and a half times the rate of White men. Treatment manuals are not linguistically designed to address the needs of these cultural groups. Latessa et al. (2013) described the limited research examining the reduction of recidivism. For example, the binary definition of recidivism limits offenders’ progression toward changing behaviors (Klingele, 2019). Additionally, recidivism often does not consider the precipitating risk factors that precede a rearrest, such as return to substance use and pro-crime peer association (Dyck et al., 2018). Furthermore, the literature may exclude the term “recidivism.” As a result, professional counselors could benefit from understanding these limitations to better serve their clients.

### 1.1 Relevant background

In the selection of variables for this study, the recidivism prevention literature was explored across multiple topics. These were (a) key definitions, (b) demographics of persons in recidivism treatment programs, (c) description of the Thinking for a Change (T4C) recidivism prevention program, (d) the efficacy of T4C, (e) description of the Cognitive Behavioral Interventions-Core Adult (CBI-CA) recidivism prevention program, (f) the efficacy of CBI, (g) keyness and recidivism, and (h) word networks and recidivism. After these points are examined, the research questions are detailed.

In this area of research, there are essential technical definitions of widely used words, such as recidivism. Within the context of criminology research, recidivism refers to the act of committing another criminal offense after being previously convicted of a crime (Klingele, 2019). Another term requiring a technical definition is reoffending. Thomas et al. (2018) defined reoffending as the act of committing antisocial and illegal behavior following a previous criminal conviction. One focus of this study was keyness, which is a linguistics term concerning differences in word usage patterns (Scott, 2022b). A precise scientific definition of keyness is found in the Measures subsection of this article. Another focus of the study was collocation, a linguistic term describing word networks (Scott, 2022a). A detailed definition appears in the Measures subsection. The last term is manualized treatment manuals—more significantly, those that meet the standards of evidence-based practices (EBP; van Wormer and Davis, 2018). EBP treatment manuals are empirically supported for addressing addictions (Miller and Rollnick, 2013). Several EBP treatment manuals that address recidivism have been developed over the decades, including CBI-CA and T4C.

U.S. government statistics reveal racial and ethnic disparities in incarceration (Carson, 2022). Granular details about these disproportionalities can be reviewed in Table 1, which details the race and ethnicity disparities when comparing U.S. prisoner population demographics (Carson, 2022) to U.S. Census Bureau (2023) data for the general population. There is a 30% decrease in the proportion of incarcerated White individuals versus the proportion of White people in the general population. Also noted is a 20% increase in the proportion of incarcerated Black individuals versus the proportion of Black people in the general population. These disproportionalities carry forward into recidivism treatment programs.

**Table 1:** Demographics of United States prisoner population.

Race/Ethnicity	Prisoner population		Total population		Diff. in %
	Count	%	Count	%	
White	356,000	31%	204,000,000	61%	−30%
Black	378,000	32%	41,000,000	12%	20%
Hispanic	273,800	24%	62,000,000	18%	5%
AI/AN	18,700	2%	3,000,000	1%	1%
Asian	14,700	1%	19,000,000	5%	−4%
Other	122,400	11%	9,000,000	3%	8%

The sources for this table were Carson (2022) and the U.S. Census Bureau (2023). AI/AN = American Indian/Alaska Native.

One prominent recidivism prevention program is T4C 4.0, which aims to empower individuals by utilizing positive behavior reinforcers. T4C was first produced in 1998 under the direction of the National Institute of Corrections. The program combines cognitive restructuring theories to help individuals gain control over their thinking. The fourth edition of the manual was released in 2016. T4C facilitators demonstrate how to effectively use cognitive self-change, social skills, and problem-solving skills (Bush et al., 2016). The T4C authors noted that each curriculum revision had made it more user-friendly (Bush et al., 2016).

There exists sound research illustrating that T4C is an effective recidivism prevention program. Lowenkamp et al. (2009) indicated a significant statistical difference between individuals who participated in the T4C program and those in control groups. Lowenkamp et al. (2009) reported that 23% of the treatment group recidivated (i.e., were rearrested for new criminal behavior), whereas 36% of the comparison group recidivated (*χ*^2^ = 3.93; *p* = 0.047). “Thus, the difference in the odds of recidivating between the control and treatment groups indicates that the control group was 1.57 (or 57%) more likely to be arrested during the follow-up” (Lowenkamp et al., 2009, pp. 142–143). Golden et al. (2006) identified a 33% reduction in the rate of new offenses among individuals in the T4C group compared to those who dropped out. T4C sustains its effectiveness through various methods of delivery.

When examining pre and posttest results, LaPlant et al. (2020) found that T4C is as effective at improving social problem-solving skills via video conference as when the curriculum is delivered in person. T4C has been provided for over two decades. LaPlant et al. (2020) continue to adjust the curriculum, which is now in its fourth edition. However, T4C is not the only treatment manual used to address criminal behavior.

Another program and treatment manual present in the recidivism prevention ecology is the CBI-CA program manual. CBI-CA is a multicomponent, cognitive-behavioral program that provides specific interventions that target criminogenic factors and needs. CBI-CA utilizes a cognitive-behavioral therapeutic approach to empower participants with coping and recovery strategies to manage risk factors (University of Cincinnati Corrections Institute, 2021). CBI-CA focuses on developing skills to assist with cognitive, social, emotional, and coping skills. The curriculum provides modifications to allow offenders with mental illness to participate, though it is not dedicated exclusively to this population. The curriculum is designed to allow for flexibility across various service settings and intervention lengths using a modified closed group format with multiple entry points. The manual has nine modules: motivational engagement, introduction to cognitive behavioral interventions, cognitive restructuring, emotional regulation, understanding behavior patterns, choosing behavior responses, problem-solving, planning for the future, and success planning (University of Cincinnati Corrections Institute, 2021). CBI-CA has designed specialized modules to address the needs of various offender populations.

There exists less evidence for CBI-CA’s effectiveness as a treatment manual. Rather than outcome studies, the CBI-CA developers have relied on the underlying outcome research from the cognitive behavioral therapy (CBT) components they selected for their program (University of Cincinnati Corrections Institute, 2021). Three key components where the CBI developers cite underlying evidence are (a) challenging irrational beliefs, (b) engaging in healthy recovery activities, and (c) addressing criminogenic needs. Regarding the challenging irrational beliefs component, Vaske et al. (2011) found that CBT programs address irrational beliefs and provide participants with effective social skills, coping skills, and problem-solving skills. There is substantial evidence of the effectiveness of CBT in addressing cognitive distortions.

In terms of engaging in healthy recovery activities, McMinn and Campbell (2017) described extra-therapeutic factors, such as thinking and behaviors, that support change outside of the counseling setting. Extra-therapeutic factors account for 40% of an individual’s psychotherapy outcomes. With CBT, most of the growth from therapy for the individual will happen outside of counseling sessions. For example, Corey (2017) stated that the behavioral techniques in CBT include homework assignments, particularly assignments that are carried out in real-life situations. CBT also aims to provide individuals with self-therapy techniques to continue applying throughout their lives outside of counseling services (Corey, 2017). CBT provides individuals with skills and coping strategies to sustain their efforts to change outside of treatment services.

Dyck et al. (2018) listed criminogenic needs: criminal history, family/marital interactions, employment/education status, peer relations, alcohol/drug problems, leisure/recreation activities, antisocial personality/behavior patterns, and pro-criminal attitudes/orientations. Latessa and Schweitzer (2020) described how CBT can address individuals’ criminogenic needs. They asserted that CBT assists individuals with restructuring their thinking, which, in turn, reduces the chances of reoffending.

Research on keyness and recidivism is limited. Partch and Dykeman’s (2019) study on linguistic composition highlights the therapeutic potential of text messages as therapeutic interventions, with one of the benefits being a decrease in recidivism rates. O’Hara and Dykeman’s (2019) described how language matters in addiction treatment. Their study was the first to compare the linguistic components of 12-step programs. These studies point to the relationship between linguistic phenomena in treatment and recidivistic behaviors.

The study of collocations is driven by Firth’s (1957) idea that “You shall know a word by the company it keeps” (p. 11). However, no research exists on collocation and recidivism. There is research on collocations for another word in criminology: rape. Tranchese (2019) examined the collocation of the word rape in the media to better understand sexualized violence. The study focused on collocation and a concordance analysis of the words “rape” and “raped” in the corpus. The study examined the collocates of rape and raped in the concordance lines to identify contextual elements that would not be obvious through a collocation analysis alone. Tranchese confirmed the top 20 lexical collocates and six semantically related words, such as the word “victims.” Blauenfeldt (2015) examined collocation patterns when comparing the discourse patterns between rapists and pedophiles. The study grouped keywords into four distinct categories and examined the concordance, word lists, and collocation patterns, such as the category “perpetrator” (Blauenfeldt, 2015). These studies highlight the significance of performing a collocation analysis on corpuses to understand how a field uses a word. Conducting collocation analyses on treatment manuals can contribute to the literature on best practices in reducing recidivistic behaviors by understanding how words are structured within these manuals.

### 1.2 Research questions

Given the aforenoted, four research questions (RQs) were created to direct this study. These RQs were

RQ1: In comparing the CBI-CA recidivism prevention program manual to the T4C recidivism prevention program manual, what words were used with greater and lesser frequency?

RQ2: In the CBI-CA manual, what is the word network of the word with the strongest positive keyness in RQ1?

RQ3: What is the word network of the word stem crim* in the CBI-CA manual?

RQ4: What is the word network of the word stem crim* in the T4C manual?

## 2 Materials and methods

### 2.1 Design

This study employed a synchronic corpus linguistics design (Brezina, 2018). There were four variables used: manual, keyness, node word, and collocates of the node word. The two corpuses were recidivism prevention program manuals. Keyness and collocation were measured on a continuous scale, while the manual and the node word were measured on a nominal scale. The unit of analysis was single words (Bjekić et al., 2014). Given the public and published nature of the data, human subject review was not required. The minimum sample size required was assessed via an *a priori* power analysis employing G*Power 3.1 (Faul et al., 2009). This study employed a chi-square derivative, with Cohen’s w used as the effect size input. The average effect size (*w* = 0.32) was secured from a recent forensics study (Elphick et al., 2021). The input parameters were (a) test family- *χ*^2^ tests; (b) statistical test- goodness-of-fit tests: contingency tables; (c) type of power analysis- a priori: compute required sample size- given *α*, power, and effect size; (d) *w* = 0.32; (e) power (1-*β* error probability) = 0.80; (f) *α* = 0.001; and (g) degrees of freedom (*Df*) = 1. The G*Power 3.1 output suggested a sample size of 167 with an actual power of 0.80.

### 2.2 Corpuses

#### 2.2.1 Overview

Three inclusion criteria were used to select the texts for the study and reference corpus. These were (1) theoretical approach, (2) manual pragmatics, and (3) assignment as a study or reference corpus.

Criterion 1 limited manual selection to those with a CBT theoretical orientation. This restriction was used because, within criminal justice, CBT reduces antisocial thinking and criminal behavior by targeting the offender’s behaviors, such as anger issues, accountability for actions, and developing problem-solving and coping skills (Lipsey et al., 2001; Vaske et al., 2011). CBT meta-analyses demonstrate reduced recidivism in the incarcerated population (Aos et al., 2006; Butler et al., 2006; Ferrito and Moore, 2017; Harrison et al., 2020; Henwood et al., 2015; Lipsey et al., 2007). CBT is considered the gold standard of psychotherapy when working with individuals in the criminal justice system (Aos et al., 2006; Butler et al., 2006; David et al., 2018; Feucht and Holt, 2016). There are various manual-based CBT curricula, so criteria were created to narrow the selection of the treatment manuals for this study.

Criterion 2 addresses manual selection pragmatics. The CBT manuals included had to be (a) in use for over 5 years, (b) widely adopted, and (c) readily available in an electronic format that could be converted to plain text. These inclusion criteria were employed because of the significance of these manual-based CBT curricula for reentry and recidivism treatment programs and because of accessibility for this study. Application of these three criteria against the known universality of CBT recidivism manuals left two: the CBI-CA (University of Cincinnati Corrections Institute, 2021) and T4C (Bush et al., 2016).

Criterion 3 for assignment as the study corpus or the references corpus comprised two corpuses. The first was CBI-CA as the study corpus. The rationale for this assignment was that CBI-CA is the more recent addition to the body of CBT curricula. Despite its increased popularity and use, little is known about how it differs from the gold standard of CBT recidivism curricula. The second was T4C as the reference corpus. The rationale for this assignment was that T4C’s longevity, wide adoption, and body of research, including diverse delivery methods, made it the ideal reference against which to understand the changes present in the newest addition to the CBT recidivism treatment manual ecology.

#### 2.2.2 Study Corpus (CBI-CA)

The register for this study was academic prose. The subregister was psychological treatment manuals. The scope and source were the CBI-CA recidivism prevention program manual (University of Cincinnati Corrections Institute, 2021). In particular, the inclusion criteria were treatment approaches considered as EBP and treatment manuals currently being utilized to address recidivism. The exclusion criteria were EPB approaches that did not have a treatment manual and EBP approaches with multiple treatment journals. Two treatment manuals were selected from the inclusion and exclusion criteria, one to be the study corpus and one to be the reference corpus. T4C met the inclusion and exclusion criteria and was selected because of its longevity and research substantiating its effectiveness since 1998 (Bush et al., 2016). CBI-CA met the inclusion and exclusion criteria and was selected because the University of Cincinnati Corrections Institute (2023), the developers of the treatment manual, are considered subject matter experts on rehabilitative services for offenders (University of Cincinnati Corrections Institute, 2023).

The size of this corpus was 23,421 words and 2,311 different word types. During preprocessing, electronic files of the manuals were converted to .txt format using AntFileConverter (Anthony, 2017). These .txt files were then cleaned for non-ASCII characters and diacritics. Stopwords, such as “the,” “of,” and “an,” serve grammatical functions but do not contribute to content meaning (Wilbur and Sirotkin, 1992). These words were removed during preprocessing using a standard list of such words (Natural Language Toolkit [NLTK] stopwords; Bleier, 2010).

#### 2.2.3 Reference Corpus (T4C)

The register and subregister were the same as for the study corpus. The scope and source were the T4C recidivism prevention program manual (Bush et al., 2016). The size of this corpus was 41,541 words and 2,911 different word types. Preprocessing was conducted in the same manner as for the study corpus.

### 2.3 Measures

#### 2.3.1 Keyness

A keyness study reflects the words that are particularly important within a corpus (Scott and Tribble, 2006). Words that frequently appear in one corpus may infrequently appear in another at a statistically significant level. As such, a keyness study identifies the most prominent and frequent words within a corpus (Jensen, 2020). A positive keyword is a word that occurs more often than would be expected by chance when compared with the reference corpus. A negative keyword is a word that occurs less often than would be expected by chance when compared with the reference corpus.

#### 2.3.2 Collocation

Collocation examines the placement or position of a word, particularly in relation to the node word within a text (Brezina et al., 2015; Gablasova et al., 2017).

#### 2.3.3 Node word

Brezina (2018) described a node as the word, phrase, or grammatical structure of interest. The node word is essential to understanding the frequency, word positioning, and the linguistic relationship between terms. Node words and their related lexical networks were selected from both treatment manuals.

### 2.4 Data analysis

For RQ1, the descriptive statistics reported include raw frequency count and normalized frequency count (count per 1,000 words). In terms of inferential analysis, differences between the corpuses were assessed using the log-likelihood ratio test (*G*^2^). This study presents statistics for the 10 words with the strongest keyness in each direction. The effect size metric employed was the log ratio (LR). When a word is twice as common in A as in B, then the binary log of the ratio is 1 (Hardie, 2014). The alpha level was set at *p* < 0.001, and the analysis was completed using the R package “textstat_keyness” (Benoit et al., 2018). Regarding RQs 2–4, the GraphColl module of #Lancsbox was used (Brezina et al., 2018). The GraphColl settings were (a) span: 5 left, 5 right; (b) statistics: 03-MI; (c) threshold: MI = 3, collocation frequency = 5; and (d) type = type; filter = stopwords. Complete keyness results are available at https://osf.io/kngzx/.

## 3 Results

**Figure 1:**
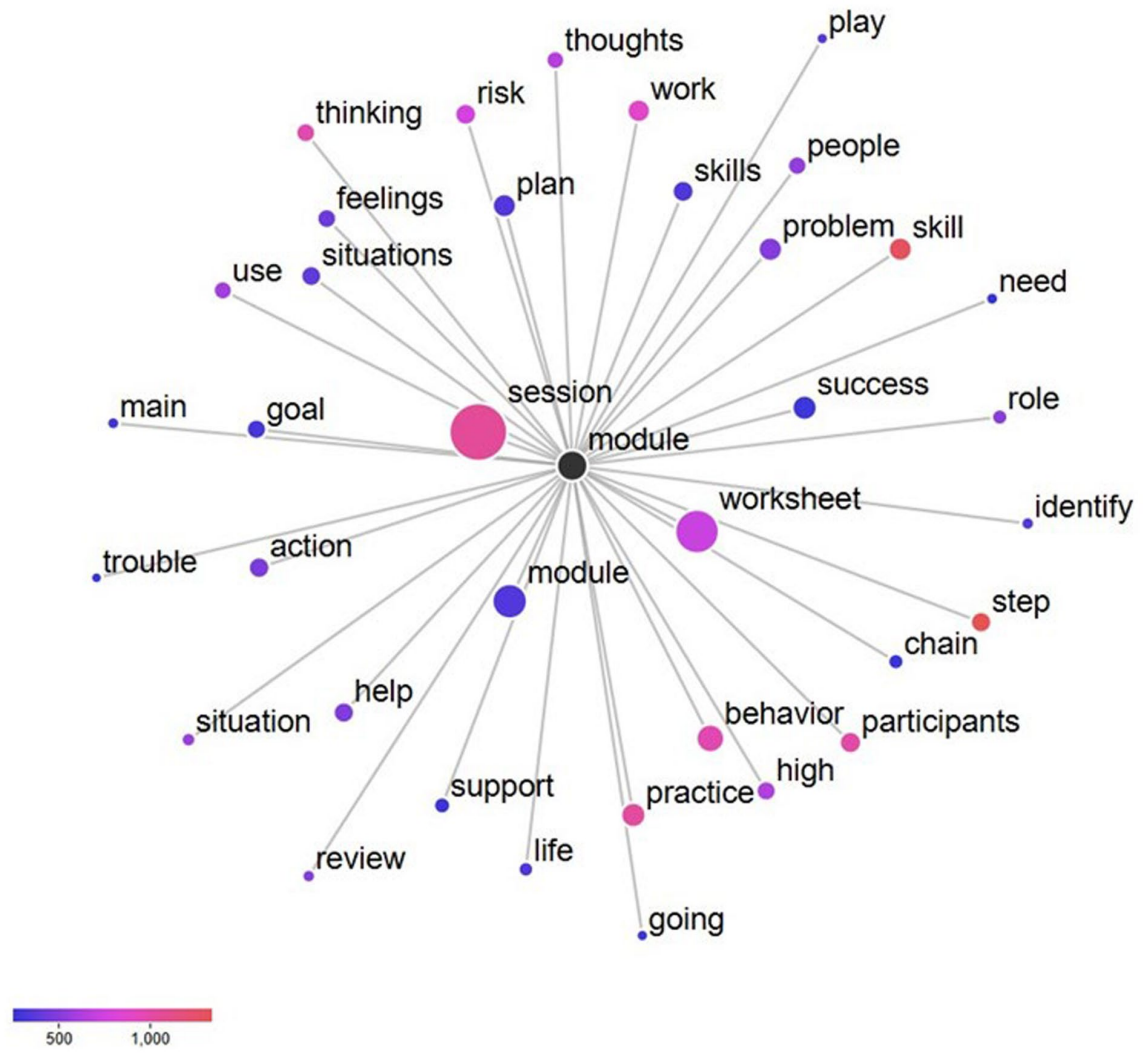
Collocates of the node “Module” in the CBI-CA manual (RQ2).

**Figure 2:**
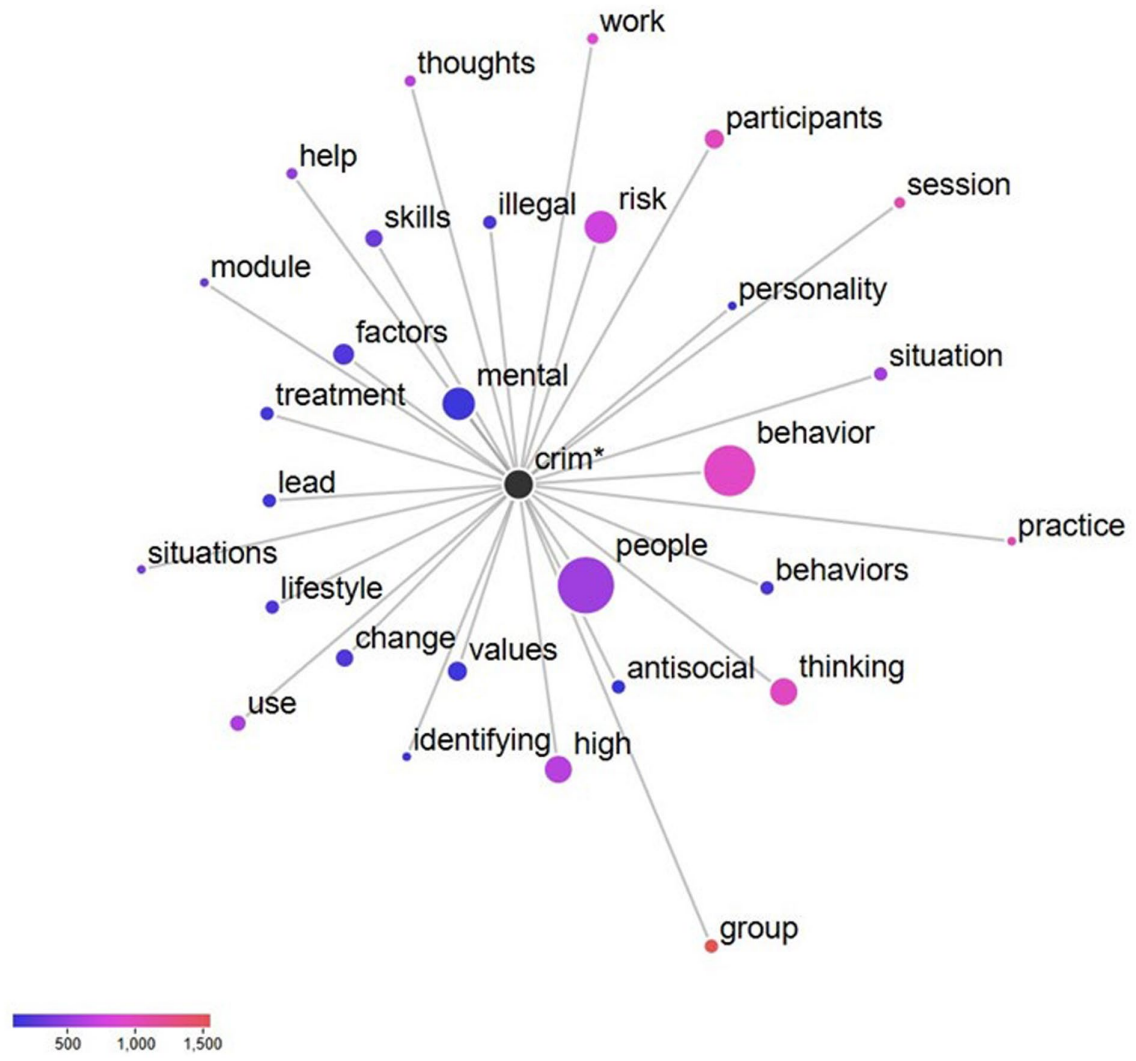
Collocates of the Node “Crim*” in the CBI-CA manual (RQ3).

**Figure 3:**
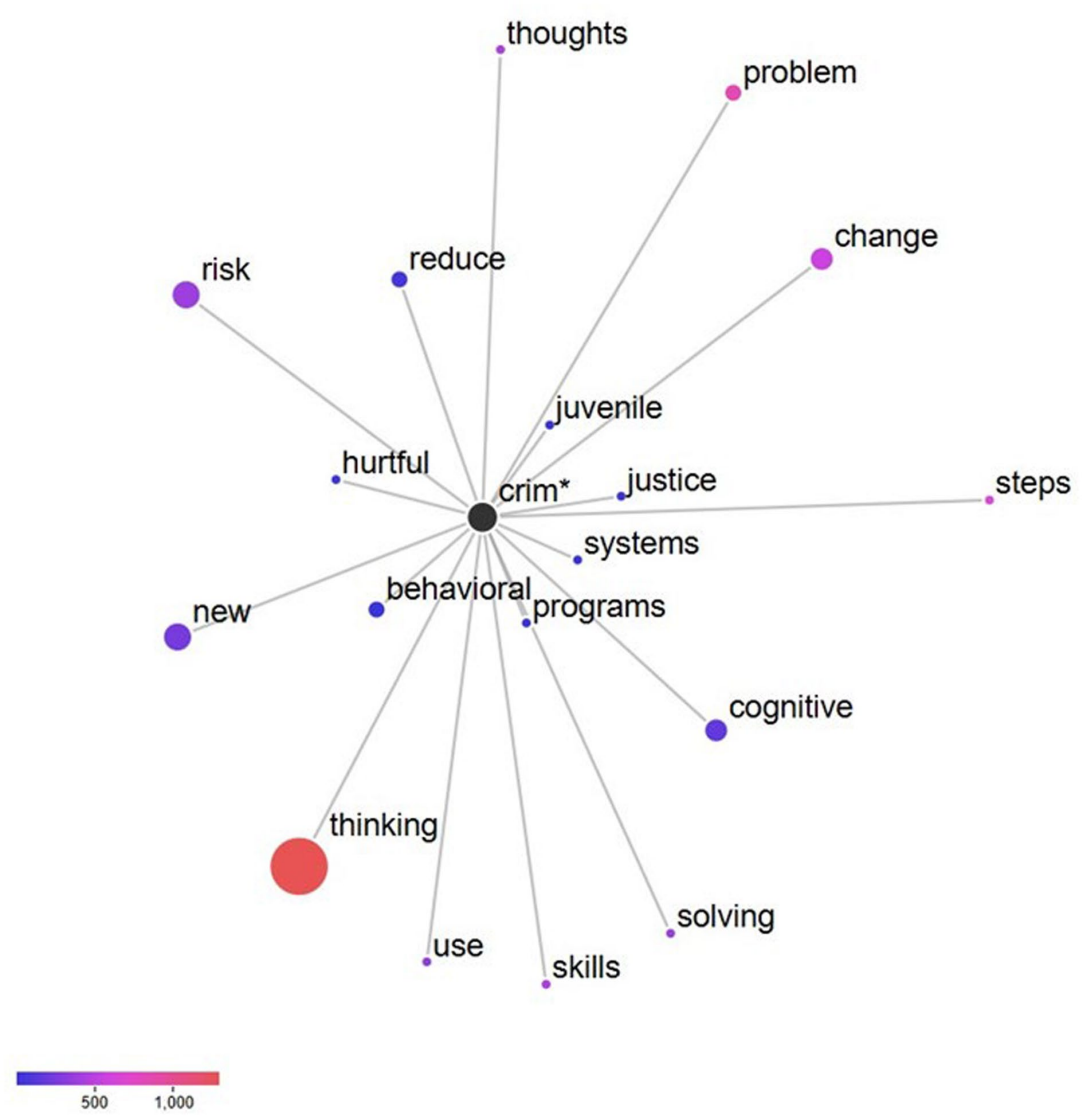
Collocates of the Node “Crim*” in the T4C manual (RQ4).

In terms of RQ1 (words occurring with greater and lesser frequency in the study corpus), the three words with the strongest positive keyness were “module,” “success,” and “worksheet.” The three words with the strongest negative keyness were “lesson,” “supplement,” and “handout.” Table 2 provides a complete list of the top 10 keywords in both directions. Regarding RQ2 (the word network of the strongest positive keyword), the most frequent term in the study corpus was “module.” This term was further analyzed to yield the strongest collocates: “session” and “worksheet.” A complete list of the strongest collocates for the keyword “module” can be found in Figure 1. Concerning RQ3 (the word network of the word stem crim* in the CBI-CA manual), the strongest collocates were “people” and “mental.” The complete word networks for the word stem crim* in the CBI-CA manual can be found in Figure 2. In reference to RQ4 (the word network of the word stem “crim*” in the T4C manual), the strongest collocates were determined to be “systems” and “justice.” The complete word networks for the word stem crim* in the T4C manual can be found in Figure 3.

**Table 2:** Keyness results (RQ1).

Direction	Word	CBI-CA	T4C	*G* ^2^	LR
Positive	Module	342	0	315.098	8.645
	Success	277	0	255.007	8.339
	Chain	255	0	234.690	8.219
	Managing	249	0	229.151	8.185
	Pointer	231	0	212.539	8.076
	Modified	201	0	184.868	7.875
	Lifestyle	200	0	183.946	7.868
	Worksheet	716	2	639.148	7.719
	Coping	172	0	158.139	7.649
	Treatment	168	0	154.454	7.615
Negative	Supplement	0	192	385.188	−9.373
	Lesson	3	789	1,562.022	−8.850
	Handout	0	120	240.454	−8.692
	Denoted	0	80	160.196	−8.106
	Bender	0	75	150.171	−8.012
	Sherry	0	67	134.135	−7.849
	Shewan	0	67	134.135	−7.849
	Appendix	0	61	122.111	−7.714
	Slides	0	60	120.107	−7.690
	Solver	0	60	120.107	−7.690

Normalized frequency is count per million. The study corpus was the CBI-CA manual, and the reference corpus was the T4C manual. The critical value for *G*^2^ at *p* < 0.001 is 10.83.

## 4 Discussion

This study sought to explore the word usage of treatment manuals used by the criminal justice systems and treatment providers to reduce recidivism. The study compared the words used more and less frequently in the CBI-CA manual to those in the T4C recidivism prevention program manual. It identified the word network of the word with the strongest positive keyness in the CBI-CA manual. Lastly, the study examined the word network of the word stem crim* in CBI-CA and T4C manuals.

### 4.1 Analysis of obtained results

Concerning RQ1 (word usage differences between manuals), two probable reasons exist for the obtained results. One explanation is that the differences reflect minor differences in the CBT approach contained within each manual—for example, CBI-CA’s heightened discourse on self-efficacy (e.g., success). An alternative explanation is that CBI-CA has a more engaging approach to word usage that reflects a less pedantic stance (e.g., lesson, supplement) than the T4C curriculum. Of these two explanations, the latter is most likely between these two explanations because CBI-CA has woven in motivational interviewing, and the word usage reflects the use of motivation engagement techniques throughout the curriculum to focus on successes and to avoid power struggles with participants.

Regarding RQ2, the most frequent term in the study corpus was “module,” and the two strongest collocates were “session” and “worksheet.” Merriam-Webster (n.d.-a) defines a module as “an educational unit which covers a single subject or topic,” a session as “a meeting or period devoted to a particular activity” (Merriam-Webster, n.d.-b), and a worksheet as “a sheet of paper on which are printed exercises and problems to be solved by a student” (Merriam-Webster, n.d.-c). There are two probable reasons for the obtained results. First, the use of these words reflects the inherent psychoeducational emphasis in any cognitive-behavioral interventions. Specifically, these words capture the CBI-CA’s guided approach to linking thoughts and behaviors, teaching individuals to identify risky thoughts, and implementing new thinking (University of Cincinnati Corrections Institute, 2021). The goal of the treatment manual is to replace the risky thoughts, feelings, and beliefs (University of Cincinnati Corrections Institute, 2021). Second, the instruction-tinged words reflect the demands by various recidivism treatment funding authorities for defined intervention outcomes. Of the two reasons, the first is most likely because treatment theory is a more probable driver of recidivism interventionists than external funding mandates.

RQ3 examined the word network of the word stem crim* in the CBI-CA manual. The most collocated words to the stem word crim* were “people” and “mental.” One explanation is that CBI-CA focuses on the therapeutic alliance and mental health needs to address the individual’s criminality. The following quote illustrates CBI-CA’s focus on counselors addressing mental health concerns within correctional institutions: “The Council of State Governments (CSG) and Bureau of Justice Assistance (BJA) provided funding to the University of Cincinnati Corrections Institute (UCCI) to develop and implement an evidence-based, cognitive-behavioral program for people with mental illnesses involved with the criminal justice system” (University of Cincinnati Corrections Institute, 2021, p. 2). An alternative explanation is that CBI-CA uses person-centered and softer language to address the criminogenic factors. Here is an example from the CBI-CA manual: “Mood is particularly transient for people living with mental illnesses involved in the criminal justice system” (University of Cincinnati Corrections Institute, 2021, Pretreatment Session 2–3). Of these two explanations, the former is most likely between these two explanations. As mentioned earlier, CBI-CA uses motivational interviewing and engagement techniques throughout the curriculum. These techniques assist the individual in recognizing their mental health and interpersonal barriers to wellness, sobriety, and a crime-free lifestyle.

RQ4 assessed the word network of the word stem crim* in the T4C manual. The most collocated words to the stem word crim* were “systems” and “justice.” The first explanation is that T4C is utilized within correctional institutions. For example, Williams and Hanley (2005) emphasized that it is detrimental for clinicians to focus on criminal thinking errors. The second explanation is that T4C uses the term for the orientation of the facilitators and the participants when describing the curriculum to individuals involved with the criminal justice system. For example, Bush et al. (2016) stated, “The work of these individuals set the foundation and benchmarks for many of the programs and cognitive behavioral curricula currently developed and implemented, including those used throughout the criminal and juvenile justice systems” (p. vii). Between these two explanations, the second is most likely because of the foundational history of T4C and its inception within the National Institute of Corrections’ (NIC) cognitive approaches to changing offender behavior training seminar.

### 4.2 Limitations

Two limitations, both related to corpus construction, should be considered when interpreting these findings. The first concerns the number of treatment manuals available for the study. For proprietary reasons, many manual publishers severely restrict access to their products. A second limitation was the availability of digital copies, where there were no direct proprietary barriers but practical ones were encountered. For example, The Change Companies^1^ offers a range of evidence-based curricula, but these materials are only available in print.^2^ As such, researchers would need to undertake a massive Optical Character Recognition (OCR) process to convert all print materials into digital form.

### 4.3 Implications

Three implications emerge from the findings of this study. First, the rigid practice fidelity standards present in extant EBP recidivism manuals restrict clinicians’ ability to adapt treatment approaches to local demands and populations. In addition, as part of EBP, many manual developers require formal facilitator training before granting access to their manuals. For example, the CBI-CA curriculum requires over 20 h of training, which includes several hours of live observation of group facilitation. T4C requires 32 h of training. Completing multiple training sessions for various treatment manuals is time-consuming. In addition, this training can cost thousands of dollars per group facilitator, which is not cost-effective for some clinicians or smaller treatment programs.

Second, many treatment manuals were unavailable for the study due to proprietary restrictions and limited digital availability. These barriers obstruct advancements in EBP treatment manuals and future corpus linguistic and comparative analyses research projects because of the access needed for corpus construction.

The final implication concerns the finding that treatment manuals do not reflect the cultures with the largest populations inside the correctional system. Future research might consider the demographics of individuals in recidivism treatment programs and the cross-cultural and linguistic attunement of the curriculum. For example, Caldwell (2016) emphasized the importance of culturally competent evidence-based treatment, noting clinicians’ responsibilities to address clients’ motivation, readiness for change, strengths, resources, and social-cultural factors. The focus on cross-cultural and linguistic attunement is an ethical responsibility given persistent racial and ethnic disproportionalities in incarceration rates. Although EBP yields better results in decreasing recidivism rates compared to treatment as usual, fine-tuning the cultural and linguistic components toward those disproportionately impacted by incarceration could enhance the facilitators’ training and the curriculum’s implementation.

## Data Availability

The original contributions presented in the study are included in the article/supplementary material, further inquiries can be directed to the corresponding author.

## Footnotes

1
https://www.changecompanies.net

2
https://www.changecompanies.net/industry/jail-and-prison.php
